# The British Warrior as He Is To-Day

**Published:** 1919-03-08

**Authors:** 


### MODERN METHODS OF WARFARE.

### The British Warrior as he is To-day.

By J. H. M.

When the British Cabinet made that momentous decision on August 3, 1914—how momentous we realise now—and when the call to arms was sounded we were not surprised to see men flocking to the Colours in thousands and tens of thousands. We felt it a fine thing that men should give up lucrative positions to fight for their country; that boat-loads of volunteers from the Overseas Dominions should hasten back to England; that men from the Argentine to China should find a response in their hearts and wills to the call; and that so many men of education and social position, not waiting for commissions, should join the ranks. But it was just what was to be expected of the British race. And when Canada, Australasia, and South Africa began to furnish their own contingents we felt that the British Empire was indeed one.

We knew that the New Army and Territorial Force would maintain the character and show the same fighting spirit which British soldiers have always possessed, and which was then being manifested again by the original Expeditionary Force in France.

Out of the raw material, drawn from every grade of society and every class of life crowding to the depôts, the formidable task presented itself of training and equipping a huge army to meet a highly-trained and prepared enemy, but in a few months an efficient fighting force was ready to take the field. The sergeants and sergeant-majors, most of whom returned to the Army from civil life, have not received sufficient credit for what they have done.

A stream of eager, confident, and cheerful troops began to cross the Channel, officered by men of that gallant spirit which we look for in the English gentleman. They relieved and supported the original Army and the Territorials who had held the line during the winter and spring, and who, with some Canadians, had broken a formidable German attack with greatly inferior forces. The fighting that had already taken place had shown that this war was like no other war. While the strategical principles of warfare might remain the same, new methods had been evolved by time, and others had been devised by an unscrupulous enemy during many years of preparation. As time went on still new means of destruction were produced by both sides till the new instruments of offence and defence seemed to make modern warfare a war of machinery.

The enemy occupied nearly all the high ground, and had his trenches protected by thick belts of barbed wire and his line defended by strong positions carefully prepared. Guns concentrated behind his lines and innumerable machine-guns made any open ground no place for loitering. Trench mortars, throwing bombs of various sizes, including an aerial torpedo which hurtled over No-man's-land like some flying monster, accentuated the discomfort and danger of a life in the trenches, already harassed by artillery fire, to which at first we could only reply but feebly.

The strain of life in the trenches was increased by the possibility, always imminent during the night, of a gas attack. This gas, let loose from cylinders, came creeping over No-man's-land like a poisonous miasma. First chlorine gas, then the more lethal phosgene gas, appeared, and as a further refinement a synthetic product called mustard gas was introduced. The first two were liberated as cloud gas, but later all three, particularly the mustard gas, had the fluids, from which the gases volatilise, enclosed in shells and fired from the gun mouth—an advantage which allowed the gas to be sent in any direction, and within limits to any distance independently of the direction of the wind. A few inhalations of any of these gases, but especially of the chlorine and mustard varieties, caused such irritation of the respiratory passages that the lungs become filled with inflammatory secretion with consequent exclusion of air, so that those who escaped an early death might become tortured with a distressing pain in the chest and difficulty in breathing. Mustard gas, in addition, from its peculiar irritating character, blinded the eyes and scorched the skin like fire.

Life in the trenches, by constantly standing in wet mud, often produced in the soldiers, and sometimes in officers, a condition called "Trench foot," in which the feet became dead to feeling, and then swollen, a state of things which might lead to ulceration and even gangrene. "Trench fever," a lingering feverish disorder, lay in wait for the occupants of the trenches, for which the rat—that constant companion of the soldier in the trenches—is indirectly responsible. That rodent harbours a parasite which is equally at home on man, and by migrating to the human occupants of the dug-outs, it is believed infected many with that new disease.

New methods of warfare, with its system of holding a line of trenches during the winter and with its machinery of destruction, required corresponding methods of protection and defence. Deep and strong dug-outs, always dark and dank, were constructed. Long rubber boots to keep the feet dry were worn. Steel helmets replaced the cap in the forward areas, and were the means of diminishing head wounds, always serious, by 50 per cent. Box respirators, a complete protection against gas, were perfected, and gas alarms had sentries whose whole duty it was to watch for a gas attack; while No-man's-land was illuminated at frequent intervals at night by flares in order to prevent a surprise attack. More and more machine-guns, a greater supply of artillery and trench mortars were needed, as well as bombs or hand grenades for fighting at close quarters. It was resource against cunning; ingenuity matched against ingenuity. For courage and brave leadership alone were of little avail, as the unfruitful year of 1915 proved. Only when we had countered the enemy's superiority in the material of war would courage prevail. At length, however,

**Modern Methods of Warfare**—(*continued*).

the organisation of the production of munitions and aeroplanes and the applied inventive genius of the British, ending in the invention of tanks, put us in a position for a successful offensive.

Stationary warfare, with its dangers and discomforts, but comparative safety, demanded patient endurance. It became stoicism, but, always alert and vigilant, was far from being an inert fatalism, though raids requiring cool daring gave opportunity for the fighting spirit of the troops to exercise itself. In a battle, however, attacking across No-man's-land, swept by machine-gun bullets, through a barrage of shells and barbed wire, perhaps imperfectly cut, in order to reach a concealed enemy, called for the utmost determination and courage. But it was movement, and there was an object to be won; and there was the excitement of grappling with an enemy. But in a battle it is not always movement against an enemy. There are positions won at great cost to be held against counter-attacks, and through the murderous fire of a bombardment when great shells which, approaching with a terrifying shriek, make the earth quake with the explosion. There are supplies of food and ammunition to be brought up under shell-fire and there are the wounded to be brought in.

In former wars battles have been short and sharp affairs, but in this war the same men have fought day after day and month after month, with only short spells of rest. In former wars wounds have been caused almost entirely by bullets, but in this war bullet wounds have been much less common than wounds caused by shell fragments: wounds such as have been rarely seen before. Large gaping wounds with flesh lacerated and bone shattered have been a common sight in the hospitals. These wounds, and, indeed, all wounds, have demanded prompt and thorough operative treatment, for otherwise they may become infected by microbes which rapidly produce gangrene of the tissues, with consequent death—it may be of a limb or even of the patient. But a man may become incapacitated during a battle or after it from other causes than wounds. The stability of men's nervous systems vary, and the terrible strain of a bombardment, the bursting of shells close at hand, burying men in a trench or tossing them in the air, often resulted in a disturbance of the fine organisation of the higher nerve centres, so that a man, if he escaped death or wounds, may have been bereft of intelligence and volition. Speechless or paralysed, he becomes as a babe, happily not irrecoverable, but many months will pass before mind and will and nervous tone recover. Others less severely affected have found the continued strain too much and have had to retire for a rest.

There are a few men who delight in danger and know not fear, but the courage of most men in face of danger arises, partly at least, from a sense of duty, or from the instinct of self-preservation, which overcomes fear. This is a courage which often rises with danger, and which, in the excitement of battle, may attain to heights of sublime heroism and self-sacrifice. And yet how many instances do we not know of cool, calculated daring in the absence of any excitement? Not less noteworthy, too, is that stubborn obstinate bravery, which, when the day goes badly, if discouraged, is not dismayed; that tenacity which holds on to a hardly-won position and which, even in defeat, waits for an opportunity to redeem itself.

The Germans said at the beginning of the war that the nation with the strongest nerve would win, and perhaps they spoke truly. Except for a few faint-hearts the spirit of Britain has never wavered and her warriors from all over the wide extent of the Empire have stood the terrible strain with that tenacity and determination that the enemy has always ascribed to the British race and which he has always feared in this war.

Race is not synonymous with nationality. A nation may be composed of different races with different characteristics, but the British are a race which, if not entirely homogeneous, has during nearly a thousand years blended into a people with the characteristics and stability of a pure race.

Race is breed with the inherited qualities of that race, and inherited qualities are slowly modified and that only in the course of several generations. The British race has still the qualities possessed at the time of Elizabeth and Cromwell, and Britain's soldiers of to-day are of the same stuff as those of Marlborough and Wellington: with a fighting spirit, dauntless and daring in attack, steady and undismayed in defeat, and always sure of winning one battle—the last.

Four years of war cannot fail to have had effects both good and bad, on those who have brought us victory. It is true that the hard life of the trenches and battlefield has had a roughening effect on some, a roughness which will be smoothed again by contact with civilisation and refinement. But the life of war has also had a hardening and strengthening effect—more permanent. It has taught the soldier self-reliance and self-control, resource in difficulties, and patience in hardship and suffering. And a period in the Army has developed the manhood of many a lad whose development would have been circumscribed by the narrow confines of his home, business or workshop.

Hardship may harden the spirit but not necessarily the heart. Many have faced death not once but often, and he who looks at death looks also into his own soul. The soldier disdains weakness, but a comrade in distress will find sympathy and tender help, and even a prisoner belonging to a ruthless and truthless foe will find kindness when the heat of battle is over.

Our noble dead have upheld the honour of Britain and have given their lives a sacrifice for justice and right. Over half a million of our best and bravest have fallen on the field of battle or died of wounds, and many more than that are maimed for life. Britain is wounded and has bled freely. But she will recover. The stock is not exhausted; it remains the same and will produce the same kind of men. And her record, always glorious, will be glorious still.

